# *Ex vivo* drug sensitivity screening in multiple myeloma identifies drug combinations that act synergistically

**DOI:** 10.1002/1878-0261.13191

**Published:** 2022-03-12

**Authors:** Mariaserena Giliberto, Deepak B. Thimiri Govinda Raj, Andrea Cremaschi, Sigrid S. Skånland, Alexandra Gade, Geir E. Tjønnfjord, Fredrik Schjesvold, Ludvig A. Munthe, Kjetil Taskén

**Affiliations:** ^1^ 6305 Department of Cancer Immunology Institute for Cancer Research Oslo University Hospital Norway; ^2^ 6305 K.G. Jebsen Centre for B Cell Malignancies Institute of Clinical Medicine University of Oslo Norway; ^3^ 6305 Centre for Molecular Medicine Norway Nordic EMBL Partnership University of Oslo Norway; ^4^ 6305 Oslo Centre for Biostatistics and Epidemiology University of Oslo Norway; ^5^ 6305 Department of Haematology and Oslo Myeloma Centre Oslo University Hospital Norway; ^6^ 6305 Department of Immunology and Transfusion Medicine Oslo University Hospital Norway; ^7^ Present address: Synthetic Nanobiotechnology and Biomachines Centre for Synthetic Biology and Precision Medicine CSIR Pretoria South Africa; ^8^ Present address: Singapore Institute for Clinical Sciences (SICS) ASTAR Singapore

**Keywords:** drug combinations, ex vivo drug sensitivity, gain(1q21), patient‐derived MM cells, precision medicine, synergy

## Abstract

The management of multiple myeloma (MM) is challenging: An assortment of available drug combinations adds complexity to treatment selection, and treatment resistance frequently develops. Given the heterogeneous nature of MM, personalized testing tools are required to identify drug sensitivities. To identify drug sensitivities in MM cells, we established a drug testing pipeline to examine *ex vivo* drug responses. MM cells from 44 patients were screened against 30 clinically relevant single agents and 44 double‐ and triple‐drug combinations. We observed variability in responses across samples. The presence of gain(1q21) was associated with low sensitivity to venetoclax, and decreased *ex vivo* responses to dexamethasone reflected the drug resistance observed in patients. Less heterogeneity and higher efficacy was detected with many combinations compared to the corresponding single agents. We identified new synergistic effects of melflufen plus panobinostat using low concentrations (0.1–10 nm and 8 nm, respectively). In agreement with clinical studies, clinically approved combinations, such as triple combination of selinexor plus bortezomib plus dexamethasone, acted synergistically, and synergies required low drug concentrations (0.1 nm bortezomib, 10 nm selinexor and 4 nm dexamethasone). In summary, our drug screening provided results within a clinically actionable 5‐day time frame and identified synergistic drug efficacies in patient‐derived MM cells that may aid future therapy choices.

## Graphical abstract

A drug sensitivity screening method allows assessment of cell viability effects after treatment with drugs alone and in combination in cancer cells from multiple myeloma patients. Differential drug sensitivities linked to corresponding *in vivo* clinical responses and synergistic drug combinations were identified. This approach may enable prediction of drug efficacies and could assist clinical decisions on future therapy choices.

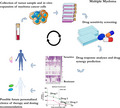

**Abbreviations**
BCL‐2: B‐cell lymphoma 2
BM: bone marrow
BMMC: bone marrow mononuclear cell
Bort: bortezomib
BTK: bruton’s tyrosine kinase
Carf: carfilzomib
CCND1: cyclin D1 gene
CR: complete response
CTG: CellTiter‐Glo
Dex: dexamethasone
DSS: drug sensitivity score
FISH: fluorescence in situ hybridization
HDAC: histone deacetylase
HDACIs: histone deacetylase inhibitors
HMG‐CoA: 3‐hydroxy‐3‐methylglutaryl‐coenzyme A reductase
IC_50_
: half maximal inhibitory concentration
IMIDs: immunomodulatory drugs
MCL‐1: myeloid cell leukemia‐1
MM: multiple myeloma
MR: minimal response
NDMM: newly diagnosed multiple myeloma
NF‐KB: nuclear factor kappa‐light‐chain‐enhancer of activated B cells
ns: not significant
PIs: proteasome inhibitors
PR: partial response
RB1: retinoblastoma protein
RMM: relapsed multiple myeloma
SD: stable disease
SMM: smoldering multiple myeloma
VGPR: very good partial response

## 1 Introduction

Multiple myeloma is an incurable malignancy of plasma cells within the bone marrow (BM). It is the second most common hematological malignancy in high‐income countries, accounting for 1% of all tumors [1]. To overcome resistance and increase response durability, MM treatment relies on drug combinations. Modern treatment combining proteasome inhibitors (PIs), such as bortezomib and the immunomodulatory drug (IMID) lenalidomide, has improved survival in MM [1, 2]. However, MM remains hard to treat successfully, and most patients require several lines of therapy, because the disease is heterogeneous and evolves over time [3, 4]. There are currently few diagnostic aids to support treatment choices for the next line of therapy. We hypothesized that an *ex vivo* drug sensitivity test could pinpoint viable options and synergistic combinations in MM. Such *ex vivo* drug‐testing approaches have come into focus as a part of precision medicine initiatives in various cancer types, and their capability of predicting disease‐specific sensitivities has been demonstrated [5, 6, 7, 8]. In MM, many different combinations of drugs are available, complicating the treatment management. This raises the need for diagnostics to aid the choice of treatment.

To address this challenge, we established an *ex vivo* drug sensitivity testing pipeline to assess sensitivity against a panel of available and clinically used drugs and tested synergistic effects of combinations of two to three drugs in patient samples.

Our approach demonstrated drug efficacies and identified synergy in clinically useful combinations and disclosed differences in sensitivity that could be linked to clinical responses. We also showed novel *ex vivo* synergistic effects between recently approved anti‐myeloma drugs such as melphalan flufenamide (melflufen) plus panobinostat. The well‐tolerated and effective combination melflufen‐dexamethasone for patients with relapsed/refractory myeloma [9] had augmented efficacy when including panobinostat.

With the increasing number of promising drug candidates undergoing clinical testing, our combinatorial testing approach may facilitate a rationale to suggest new drug combinations in MM, while at the same time supporting individualized treatment choices for patients with limited therapeutic options.

## 2 Materials and methods

### 2.1 Study approval

Bone marrow samples from MM patients at diagnosis or at relapse were procured from the Oslo Myeloma Centre at Oslo University Hospital and used fresh. The study was approved by the Regional Committee for Medical and Health Research Ethics for South East Norway (REC#2016/947), and patients provided written informed consent in compliance with the Declaration of Helsinki. Patient clinical data are listed in Table S1.

### 2.2 Patient samples

Bone marrow mononuclear cells (BMMCs) were isolated by Lymphoprep (Stemcell Technologies, Cambridge, UK). After removal of CD8^+^ cells (#11147D, Thermo Fisher Scientific, Waltham, MA, USA), BMMCs without isolation of CD138^+^ MM cells were stimulated by CD3/CD28 (#11132D, Life Technologies, Carlsbad, CA, USA) and 100 U·mL^−1^ human interleukin‐2 (Roche Applied Science, Penzberg, Germany) in RPMI‐1640 medium (Thermo Fisher Scientific) supplemented with 2 mm L‐glutamine (Sigma‐Aldrich, Saint‐Louis, MO, USA), 10% fetal bovine serum, 1 µm sodium pyruvate, 1% penicillin and streptomycin, hereafter termed RPMI. This strategy results in activated CD4^+^ T helper cells (Th cells) as described [10]. At 48 h, CD138^+^ MM cells were enriched by immunomagnetic beads (Miltenyi Biotec, Bergisch Gladbach, Germany, #130‐051‐301) and transferred (5000 cells per well in 25 µL volume per well) into drug coated 384‐well TC‐microplates (Greiner Bio‐One Gmbh, Kremsmünster, Austria, #781098) using an automatic dispenser (Certus Flex) (Fritz Gyger, Thun, Switzerland) (see also Executable Step‐by‐Step Protocol below).

Multiple myeloma cell purity was assessed for CD138‐PE (#MI15, Biolegend, San Diego, CA, USA), CD38‐BV321 (#HIT2, Biolegend), and CD56‐PeCy7 (#NCAM16.2, BD Biosciences, San Jose, CA, USA) conjugated antibodies by flow cytometry BD LSR Fortessa. Plates were precoated with drugs with an acoustic dispenser (Echo 550, LabCyte Inc., San Jose, CA, USA). All cells were cultured at 37 °C in a humidified atmosphere containing 5% CO_2_. DMSO (0.1%) vehicle and benzethonium chloride (BzCl) (100 µm) were negative and positive controls. At 72 h, cell viability was assessed by the CellTiter‐Glo (CTG) luminescence ATP assay (Promega, Madison, WI, USA) according to the manufacturer’s recommendations and luminescence measured with Envision Xcite plate reader (Perkin Elmer, Waltham, MA, USA). The viability of the MM cell line SK‐MM2 [11] was assessed using CellTiter‐Glo. In addition, SK‐MM2 cell proliferation and cell death were assessed as end‐point measurements and recorded at 1 and 72 h using CellTiter 96 AQueous One Solution Cell Proliferation Assay (Promega) and CellTox‐Green Cytotoxicity Assay (Promega), respectively.

### 2.3 Executable step‐by‐step protocol for preparing patient‐derived multiple myeloma cells for drug sensitivity screening

1. *Isolation of bone marrow mononuclear cells (BMMCs) from multiple myeloma (MM) patient bone marrow (BM) samples*

NOTE: Sections 1–4 should be performed under sterile conditions in a tissue culture hood. BM samples procured from MM patients should be processed the same day.

1. Pipette the BM gently up and down with a 10 mL pipette to remove clumps and filter the sample through a sterile 70 μm nylon filter into a 50 mL tube. Wash the filter once with 5 mL phosphate‐buffered saline (PBS)
2. Dilute the BM 1 : 1 with PBS
3. Split the cell suspension equally into two 50 mL tubes
4. Carefully layer 10 mL density gradient medium (Lymphoprep, Stemcell Technologies) to the bottom of the tube using a 10 mL pipette
5. Centrifuge for 25 min at 800 ***g*** at room temperature, without break. The BMMCs are now visible on top of the density gradient medium layer
6. Transfer the cells into two new 50 mL tubes using a Pasteur pipette
7. Wash with PBS by filling up the tube to 40–45 mL
8. Centrifuge for 15 min at 300 ***g***
9. Wash with PBS by filling up the tube to 40–45 mL
10. Centrifuge for 10 min at 300 ***g***
11. Resuspend the cell pellet in PBS

2. *Removal of CD8 cells*

After BMMC isolation, CD8^+^ cells are removed by addition of CD8 magnetic beads coated with anti‐CD8 antibody (Dynabeads #11147D, Thermo Fisher Scientific) following the manufacturer’s protocol.

1. Count the BMMCs
2. Centrifuge the BMMCs for 5 min at 300 ***g***
3. Resuspend the pellet in MACS buffer (1 mL per 1 × 10^7^ cells) and incubate with Dynabeads CD8 (25 μL per 1 × 10^7^ cells) for 30 min at 2–8 °C in the dark with gentle rotation
4. Place the tube in a magnetic rack for 1–2 min to remove bead‐bound CD8^+^ cells
5. Transfer the supernatant to a new tube
6. Centrifuge the cells for 5 min at 300 ***g***
7. Resuspend the pellet to a final concentration of 0.5–1 × 10^6^ cells·mL^−1^ with RPMI supplemented with 2 mm L‐glutamine, 10% fetal bovine serum, 1 µm sodium pyruvate, 1% penicillin and streptomycin (hereafter referred to as RPMI)

3. *Stimulation of MM cells*

Following isolation, the MM cells are stimulated with a T‐cell expansion cocktail.

1. Culture the CD8‐depleted BMMCs (0.5–1 × 10^6^ cells·mL^−1^) for 48 h at 37 °C in RPMI supplemented with human rIL‐2 (100 U·mL^−1^) and human T cell activator CD3/CD28 magnetic beads (25 μL per 1 × 10^6^ T cells, Dynabeads #11132D) according to manufacturer’s instructions.

4. *CD138^+^ MM cell enrichment*

After stimulation, MM cells are enriched by the use of CD138‐MACS magnetic beads (Miltenyi Biotec, #130‐051‐301) according to the manufacturer’s instructions.

1. Transfer the cells to a tube and place it in a magnetic rack for 1–2 min to remove bead‐bound T cells
2. Transfer the supernatant to a new tube
3. Count the cells
4. Centrifuge the cells for 5 min at 300 g
5. Resuspend the pellet in MACS buffer (80 μL per 2 × 10^7^ total cells) with CD138‐MACS magnetic beads (20 μL per 2 × 10^7^ total cells) and incubate for 15 min at 2–8 °C in the dark with gentle rotation
6. Place an LS MACS column (Miltenyi Biotec #130‐042‐401) onto a magnetic rack and wash once by adding 3 mL MACS buffer according to protocol. Let the MACS buffer run through
7. Place a tube below the empty LS column
8. Transfer the cell suspension to the LS column. Collect the run‐through in the tube
9. Wash the LS column three times with 1 mL MACS buffer. Collect the run‐through in the same tube (CD138^−^ cells)
10. Replace the collection tube with a new tube
11. Add 5 mL MACS buffer to the LS column. Flush out the bead‐bound CD138^+^ MM cells by pushing a plunger into the column
12. Centrifuge the collected cells for 5 min at 300 ***g***
13. Resuspend the cell pellet in 1 mL RPMI
14. Count the cells

5. *Dispensing of cells into assay plates*

1. Resuspend the cells in RPMI to a final concentration of 2 × 10^5^ cells·mL^−1^
2. Transfer 25 μL of cell suspension/well of a 384‐well assay plate to obtain 5000 cells per well

### 2.4 MM cell lines and apoptosis assay

The MM cell lines (JJN3, U‐266) were kindly provided by the Department of Clinical and Molecular Medicine, Trondheim, Norway. The cells were cultured in RPMI medium. For apoptosis assay, the MM cell line JJN3 was exposed to drugs or controls (DMSO 0.1%, 100 µm BzCl, 1 µm Staurosporine). After 72 h, samples were stained with anti‐cleaved caspase‐3 and anti‐cleaved PARP (Alexa‐647‐conjugated). Subsequently, samples were run on a BD FACS Canto II and analyzed by Cytobank as described [12].

### 2.5 Drug library and drug screening analysis

A drug library of 30 approved or investigational drugs for MM was tested for single drug efficacy at 6 concentrations over a clinically relevant range from 0.1 nm to 10 000 nm (*n* = 44) (Table S2).

Drug combinations (*n* = 19 double combinations; *n* = 25 triple combinations) were selected according to clinical importance in MM and were tested on 13 patient samples (Tables S3 and S4). Combinations were tested with a priming drug, usually the most potent drug, at its fixed IC_20_ concentration. Determination of IC_20_ (average) was based on single drug sensitivity data after optimal curve fitting and outlier removal. To test the effect of double drug combinations, the priming drug was combined with a less potent drug tested at 5 concentrations (0.1–1000 nm). For triple‐drug combinations, we used a 4‐by‐4 full concentration matrix of two drugs, each tested at 4 concentrations (0.1–100 nm) combined with a third drug at its fixed IC_20_ concentration.

### 2.6 Drug screening data analysis

A quality control assessment, including calculation of z‐prime, was computed for each plate used in the screening analysis and found to have a mean value higher than 0.6 ± standard deviation (SD) 0.18 (*n* = 94). In addition, manual curation and quality integration of data was performed for each drug plate which would take out systematic errors and outliers and improve the Z’. Relative percentage (%) of cell viability was calculated by normalizing to negative and positive control wells. Curve‐fits of normalized concentration–response data used the function drm from the r package drc [13] with the four parameter log‐logistic model, LL.4, or the logistic model, L.4, where LL.4 failed to converge. Curve fit parameters were then used to derive IC_20,_ IC_50_ and drug sensitivity score (DSS).

Drug sensitivity score values were calculated using a modified version of the DSS function available in the R package ‘DSS’ [14]. In this modified function, DSS type 1 was used, without the term for division by the logarithm of the upper limit.

For double combinations, one DSS value was calculated and compared with DSS of the single drug concentration–response curves over the same considered concentration range as used in the combination. For triple combinations, DSS values were calculated for each concentration–response curve in the matrix and averaged to give a DSS score for the combination. Unsupervised clustering of the DSS values for single drugs on MM patient samples used Euclidean distance and Ward linkage method and plotted by clustvis tool [15].

The Bliss prediction model was applied for synergy analyses [16, 17]. Visualization of synergy score was done using the synergyfinder tool on viability data transformed to inhibition data [18]. To summarize the synergy scores from triple combinations, the synergy sum was calculated over the 4‐by‐4 full concentration matrix as reported [19].

### 2.7 Statistics

Data analysis was performed with rstudio (version 3.4.4) [20], knime software (AG, Zurich, Switzerland), and graphpad prism 7 (San Diego, CA, USA). To compare two means, the Mann–Whitney *U*‐test was used; when comparing three or more means, one‐way ANOVA with Holm–Sidak's multiple comparison test or an unpaired multiple *t*‐test were applied, as indicated in the respective figure legends.

## 3 RESULTS

### 3.1 Drug sensitivity screening of MM cells

**Fig. 1:**
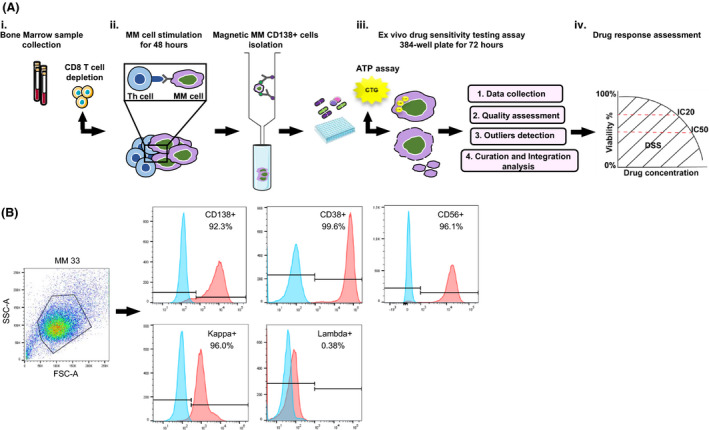
*Ex vivo* drug sensitivity screening pipeline employed for MM patient samples. (A) Workflow starting with the collection of freshly isolated bone marrow (BM) samples from relapsed (*n* = 34), newly diagnosed (*n* = 8), and smoldering myeloma (*n* = 2) patients (i), followed by depletion of CD8^+^ cells, short‐term *ex vivo* stimulation of bone marrow mononuclear cells (BMMCs) (48 h) and subsequent positive isolation of CD138^+^ MM cells (ii). Cells were transferred to pre‐printed 384‐well plates containing single drugs or drug combinations at different concentrations (iii) (see ‘Materials and methods’). After 72 h, cell viability was measured by CellTiter‐Glo luminescence ATP assay, multiple concentration–response curves extracted and overall measures of response calculated (IC_50_, IC_20_, DSS score). Prior to fitting a concentration–response curve, a quality check and data integration of raw viability reads was performed for each plate (iv). (B) Purity of MM cells used for the drug screening assays was assessed by flow cytometry with surface myeloma markers (CD138, CD38, CD56). Histogram plots from a representative patient sample (MM33) of CD138^+^, CD38^+^, CD56^+^, MM cells are shown (purity > 90%). Gates are based on unstained negative control (blue histograms).

Challenges with establishing drug sensitivity screening in MM include the *ex vivo* culturing of patient‐derived MM cells and the design of a clinically informative drug library, while accommodating a limited number of myeloma cells available for testing. MM cell growth in *ex vivo* cultures is supported by the BM microenvironment [10]. We established an *ex vivo* pipeline for cancer drug sensitivity screening by adopting our previously reported culture set‐up to ensure MM cell survival, followed by CD138^+^ MM cell isolation at 48 h (Fig. 1A i–ii).

A custom‐designed myeloma drug library (Tables S2–S4) allowed screening of limited material. To quantify and interpret drug effects across samples, ATP‐based viability readouts and drug sensitivity scores (DSS) [14] were recorded at 72 h (Fig. 1A iii–iv). Staining with myeloma cell markers CD138, CD38, CD56, and intracellular markers for κ and λ showed high purity of the MM cells (Fig. 1B).

To test the reproducibility of the *ex vivo* pipeline, we first profiled 30 single drugs (Table S2) in the MM cell lines JJN3 and U‐266 [21, 22]. We detected high reproducibility between technical and biological replicates as indicated by correlation coefficients (*R* ^2^ = 0.98–0.94 and 0.82–0.96, respectively) (Fig. S1A,B). High correlation was also observed between replicate repeats in patient samples (*R* ^2^ = 0.99) (Fig. S1C). In the drug screening assay, we found that JJN3 cells were sensitive to doxorubicin and resistant to lenalidomide (Fig. S1D). To assess consistency of the drug screening assay by an independent method, we measured the level of two apoptotic markers, cleaved caspase‐3 and cleaved‐PARP (poly‐ADP ribose polymerase) by flow cytometry in the MM cell line JJN3 in response to treatment with these drugs (72 h) and confirmed sensitivity to doxorubicin and resistance to lenalidomide (Fig. S1D,E). With these culture conditions, we had sufficient numbers of viable CD138^+^ MM cells for the drug sensitivity screening assay. Viability of MM cells could be maintained for up to 120 h, after 48 h of stimulation (Fig. S2A), in line with our observations and Wang et al. [10]. While a more moderate growth can be seen for some samples, MM cells from patients with progressive disease could be highly proliferative for up to 72 h (MM36) (Fig. S2B). When comparing the CTG assay with other methods, the readout from the CTG assay showed an increase in signal from 1 to 72 h, indicating proliferation of the SK‐MM2 cells, similarly to the readout from other viability measurement assays tested (Fig. S2C,D).

Taken together, these results show that the drug screening pipeline is robust and our *ex vivo* culture set‐up could preserve the viability of patient‐derived MM cells (CD138^+^) when stimulation was introduced for 24–48 h prior to the drug screening analyses.

### 3.2 Drug sensitivity profiling of patient MM cells reveals differential responses to conventional and novel anti‐myeloma therapies

**Fig. 2:**
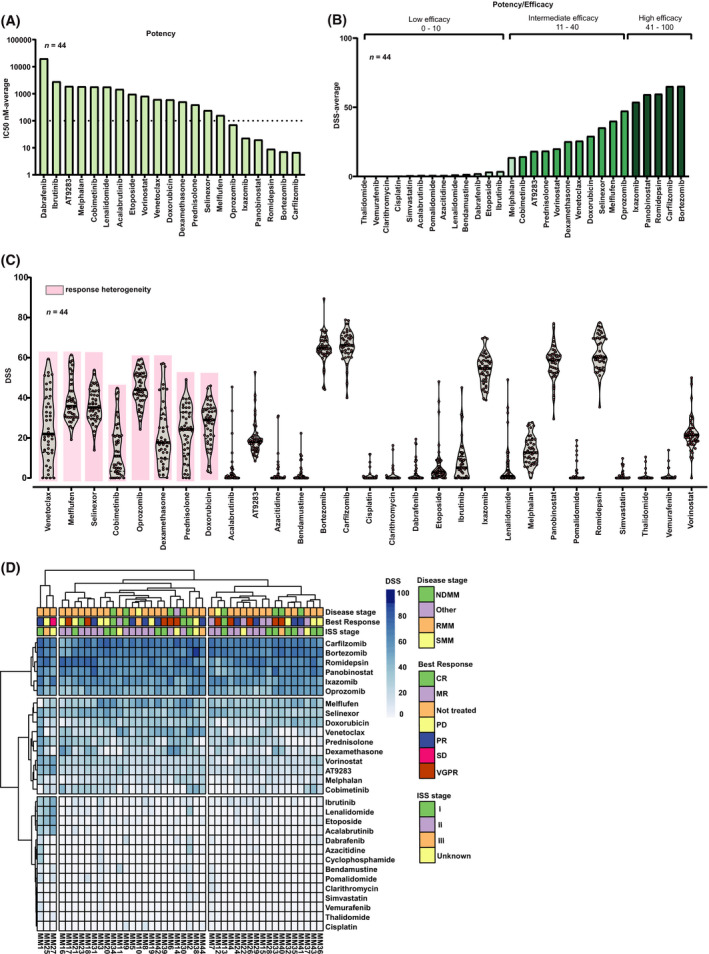
Single drug testing and response variation in MM patient samples. (A–D) Drug sensitivity assays were performed on purified CD138^+^ MM cells enriched from bone marrow mononuclear cells (BMMCs) (*n* = 44) with prior stimulation of the MM fraction as described in the ‘Materials and methods’ section. CD138^+^ MM cells were plated into 384‐well plates (5000 cells per well) and incubated with a library of 30 anti‐myeloma drugs (0.1–10 000 nm) for 72 h. Subsequently, cell viability was measured by CellTiter‐Glo luminescence ATP assay. (A) Bar plot shows estimated average of IC_50_ values. Drugs were ranked based on the IC_50_ estimates across all samples. The dotted line indicates a cut‐off set at 100 nm. (B) Bar plot shows drug sensitivity score (DSS) averages estimated from all samples. Drug responses were classified into three groups corresponding to high (dark green; mean DSS range of 41–100), intermediate (green; mean DSS range 11–40), and low efficacy (light green; mean DSS range 0–10). (C) Distribution of drug sensitivity scores (DSS) for each patient sample and with the entire drug collection. Floating violin plots indicate the median DSS (dots, *n* = 44). Pink rectangles indicate drugs with distinct heterogeneity of responses across samples. (D) Unsupervised hierarchical clustering analysis based on drug sensitivity scores (DSS) and clinical annotations of MM samples (*n* = 44) presented as a heatmap. The plot shows DSS scores for 30 drugs. Columns represent MM patient sample subgroups and rows represent drugs. Color annotations at the top (see legend, right) show disease stages (NDMM = newly diagnosed multiple myeloma, RMM = relapsed multiple myeloma, SMM = smoldering multiple myeloma), best response assessment to treatment after sampling (CR = complete response, VGPR = very good partial response, PR = partial response, MR = minimal response, SD = stable disease, PD = progressive disease), ISS stage (International staging system for multiple myeloma). Dark blue colors indicate high DSS scores (cells drug‐sensitive), and light blue colors indicate low DSS scores (cells drug‐resistant).

To determine concentration‐dependent effects of individual drugs and guide combination studies, MM cells from 44 patients were profiled against 30 drugs, and 72 h viability drug responses were evaluated. The most potent drugs with an IC_50_ < 100 nm and average DSS > 40 were proteasome inhibitors (PIs; i.e., bortezomib, carfilzomib, ixazomib, and oprozomib) and histone deacetylase inhibitors (HDACIs; i.e., romidepsin and panobinostat), followed by the newly approved agents melflufen and selinexor (IC_50_ > 100 nm; DSS 40 and 35, respectively) (Fig. 2A,B). Heterogeneous responses were noted across samples for venetoclax, melflufen, selinexor, cobimetinib, oprozomib, dexamethasone, prednisolone, and doxorubicin (Fig. 2C pink). Comparison between IC_50_ and DSS values showed high degree of consistency. However, the DSS captures both potency and efficacy of the drug [14, 23], providing a more robust measure of the drug efficacy. We therefore considered the DSS metric for downstream analyses.

To evaluate the differential drug responses further, we clustered drug sensitivities (Fig. 2D, rows) and patients (*n* = 44) (Fig. 2D, columns). We identified three drug clusters and three main clusters of patients. In cluster 1 (Fig. 2D, top row), PIs and HDACIs were found to be the most potent inhibitors across all samples, consistent with their clinical relevance in MM [24, 25]. Among the PIs, patient cells were more sensitive to bortezomib and carfilzomib (median IC_50_ 5.60 nm and 3.11; median DSS 64.5 and 66.2, respectively) than to ixazomib and oprozomib (median IC50 17.4 and 45.2 nm; median DSS 54.6 and 44.1, respectively). Of the PIs, oprozomib was the least effective (median DSS 40).

Cluster 2 (Fig. 2D, middle row) displayed the most variable drug efficacy across samples (DSS 40–10). Drugs in this cluster included dexamethasone, melflufen, melphalan, prednisolone, and venetoclax. Melflufen with a median DSS of 36 and IC_50_ around 100 nm was as expected overall more potent than melphalan (IC_50_ > 1000 nm) (median DSS 13) [26]. The commonly used dexamethasone showed differential sensitivity across samples, with DSS values ranging from DSS < 5 (16%) to a DSS > 50 (7%).

All fourteen drugs included in the last cluster (Fig. 2D, bottom row) showed low efficacy (average DSS < 10) including the negative control unmetabolized cyclophosphamide. No obvious association between patient sample subgroups and patient clinical data was found (e.g., disease stage, best response and ISS stage) (Table S1) (Fig. 2D).

### 3.3 Toward a combinatorial drug screening pipeline for patient MM cells

**Fig. 3:**
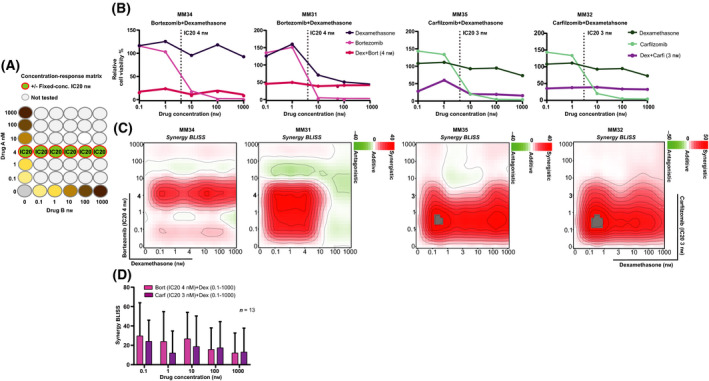
*Ex vivo* anti‐myeloma effects of drug combinations and predicted synergies. (A) Illustration of the concentration‐matrix used to test selected anti‐myeloma drugs in double combinations. The MM cells isolated from BM samples (*n* = 13) were exposed to drugs used alone and in double combinations at indicated concentrations, followed by viability testing as in Fig 2 and examples of responses shown here. Here, the first drug (drug A) is used at a fixed priming concentration while the other drug is tested at multiple concentrations (0.1–1000 nm drug B). (B) Representative concentration–response curves for the effect on MM cell viability (72 h) with the combination dexamethasone plus bortezomib (4 nm) or carfilzomib (3 nm) (IC20, dotted vertical line at 4 and 3 nm, respectively). The viability plots indicate the enhanced efficacy and potency of the tested combination compared to the single drugs. (C) Examples of 2D synergy contour plots (Bliss method) from *n* = 3 patient samples indicate areas of expected synergy (red) and antagonism (green) with the applied concentration matrix for the combination treatment dexamethasone plus bortezomib or carfilzomib. (D) Synergy score (Bliss method) across concentrations tested for all the MM patient samples (*n* = 13). Bars show mean with error bar indicating + standard deviation (SD). Combinations with a Bliss score > 0 were considered to be synergistic.

Multiple myeloma treatment relies on multiple drug combinations [27, 28]. However, analysis of synergistic effects of anti‐myeloma drugs on patient cells is largely missing in the literature. Therefore, we proceeded to test effects of combinations of two to three drugs (Tables S3 and S4). Given the limited material available 25 clinically relevant combinations were evaluated [29]. To save cells, the activity of two‐drug combinations were investigated with a priming drug at its fixed IC_20_ concentration combined with a drug at five concentrations (0.1–1000 nm) (for assay setup, see Fig. 3A).

Combinations of bortezomib or carfilzomib plus dexamethasone had striking viability effects on MM cells when compared to single drugs (Fig. 3B, as shown in patients MM34, MM31, MM35, and MM32). Furthermore, we found strong synergies (Bliss model) for these combinations, and the highest synergy was found at low concentrations, at or below 1 nm dexamethasone (mean synergy Bliss score > 15) (Fig. 3C,D).

**Fig. 4:**
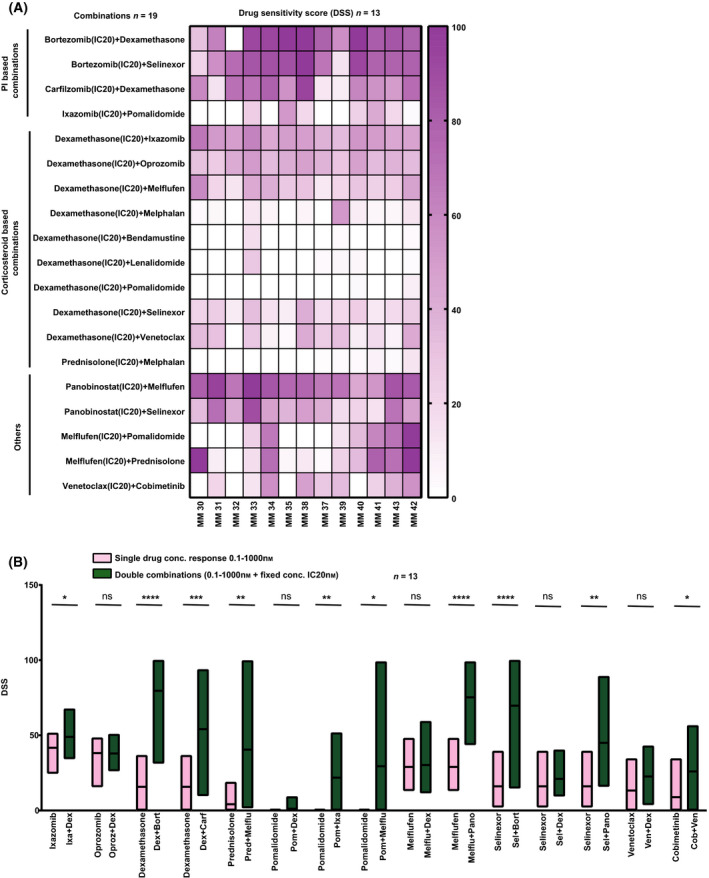
*Ex vivo* effects of clinically relevant anti‐myeloma double combinations. (A) Aggregated data showing drug effects on MM cell viability represented as DSS for 19 double combinations. High DSS indicates high sensitivity. (B) Comparison of drug efficacies between single drugs and double drug combinations with a priming drug (as in Fig 3) in MM samples (*n* = 13). Floating bar plots show min and max DSS for 25–75% confidence interval and with a line at mean. Significant differences are denoted with an asterisk (*denotes *P* < 0.05; **P < 0.01; ***P < 0.0001; ****P < 0.00001) and calculated using an unpaired multiple *t*‐test.

A heatmap of patient sample sensitivities to the 19 evaluated double combinations showed that PI‐based combinations of bortezomib or carfilzomib, with dexamethasone (median DSS 80.5 and 57.6, respectively) and bortezomib plus selinexor (median DSS 78.0) were generally effective across patient samples and with lower heterogeneity than seen for single drugs (Fig. 4A).

Our data confirmed that combinations of bortezomib or carfilzomib plus dexamethasone are effective in MM, in agreement with recent clinical results [29, 30]. Furthermore, several clinical studies have shown good results for combinations of dexamethasone with recently approved anti‐myeloma agents such as ixazomib [31], melflufen [9], oprozomib [32], selinexor [33], or venetoclax [34]. In our screen, when a fixed concentration of dexamethasone (4 nm) was combined with ixazomib and oprozomib, it yielded a high efficacy across patients (median DSS 49.0 and 37.8, respectively), compared with other corticosteroid‐based combinations. In contrast, combinations of dexamethasone with melflufen, selinexor, or venetoclax all showed intermediate efficacy (median DSS < 30). Interestingly, we experienced that one patient, MM33, whose MM cells showed very high *ex vivo* sensitivity to dexamethasone plus ixazomib (DSS = 62.19) had achieved a very good partial response (VGPR) *in vivo* (Table S1) (Fig. 4A).

Finally, comparisons of DSS between single drugs and the corresponding combinations indicated that MM cells were collectively more sensitive to combination treatments (Fig. 4B). These findings demonstrate that our approach can identify optimal synergistic drug concentrations.

### 3.4 Melflufen plus panobinostat combination shows synergistic effects in MM cells

The HDACI panobinostat has recently been approved in patients with refractory MM [35]. It is known that the combination of HDACIs with DNA‐damaging agents, inhibits cell growth synergistically [36] due to increased chromatin accessibility and reduction of DNA repair enzymes. Although the combination of HDACIs with DNA‐damaging agents can be efficacious, it can also cause unacceptable toxicity, as reported for the combination of panobinostat with the alkylating drug, melphalan (NCT00743288) [37]. We hypothesized that the combination of panobinostat with a more potent alkylating peptide drug conjugate, melflufen, [26] would provide an opportunity to improve efficacy and reduce the dose in order to mitigate intolerability in patients. Moreover, melflufen was combined with other used anti‐myeloma drugs, such as dexamethasone, prednisolone, or pomalidomide. Among these combinations, the most effective was the double combination of melflufen with panobinostat fixed at IC_20_ = 8 nm (Fig. 4A).

**Fig. 5:**
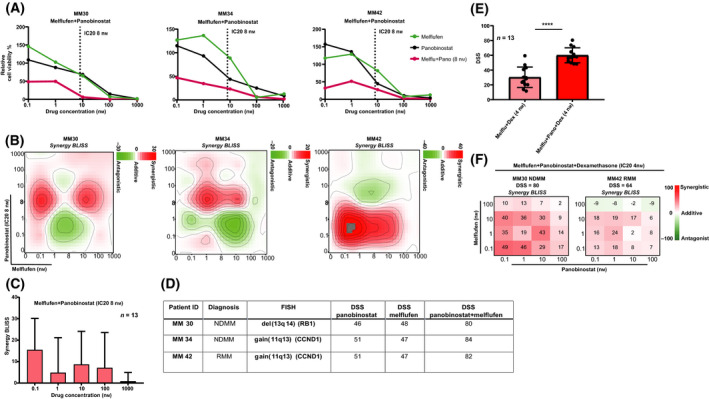
*Ex vivo* synergistic effects for the combination of melflufen plus panobinostat on MM cell viability. (A) Drug sensitivity assays with single drugs and combinations were performed on purified CD138^+^ MM cells enriched from bone marrow mononuclear cells (BMMCs) (*n* = 13) with prior stimulation of the MM fraction as described in the ‘Materials and methods’ section. Examples of concentration–response curves from *n* = 3 patient samples for the effect on MM cell viability (72 h) with the combination melflufen plus panobinostat (8 nm) (IC20, dotted vertical line at 8 nm). Viability plots show the predicted efficacy and potency of the tested combination compared to single drugs melflufen or panobinostat. (B) Examples of 2D synergy contour plots (Bliss method) from *n* = 3 patient samples (as in A) indicate areas of expected synergy (red) and antagonism (green) for the combination of melflufen plus panobinostat. (C) Synergy score (Bliss method) for the combination effects of melflufen plus panobinostat across concentrations tested for all the MM patient samples (*n* = 13). Mean + standard deviation (SD) is shown. (D) Cytogenetic data (FISH) and DSS scores for the MM samples (as in A) showing high sensitivity to the combination melflufen plus panobinostat. (E) Bar plot showing comparison of drug sensitivity (DSS) for *n* = 13 patient samples between the double combination melflufen plus dexamethasone (4 nm) and the triplet melflufen plus panobinostat plus dexamethasone (4 nm). Mean ± SD is shown along with individual data. Significance was calculated using Mann–Whitney *U*‐test (**** denote *P* < 0.0001). (F) Examples of 2D synergy contour plots (Bliss method) from *n* = 2 patient samples indicate predicted synergistic (red) and antagonistic (green) effects for the triplet on MM cell viability for patient samples MM30 and MM42. Combinations with a Bliss score > 0 were considered synergistic.

The combination melflufen plus panobinostat showed notable MM cell killing effects and increased sensitivity (mean DSS 75.3) when compared to single drug melflufen or panobinostat (mean DSS = 38.6 and 57.2, respectively) (Figs 4B and 5A). Synergistic effects on MM cell viability were also detected, and importantly, these synergies were achieved with low concentrations of melflufen (0.1–10 nm) plus 8 nm panobinostat (Fig. 5B). In general, similar patterns of synergy were observed in 11/13 patient samples (Fig. 5C). Intriguingly, we noted striking synergistic effects in patient samples MM30, MM34, and MM42 (Fig. 5D), that had mutations suggesting impaired cell cycle control such as gain(11q13) affecting the oncogene cyclin D1 (CCND1) [38] and del(13q14), giving loss of the retinoblastoma protein (RB1) [39]. On total, synergistic responses were associated with such mutations in 8 of 13 samples.

The addition of dexamethasone (IC_20_ 4 nm) to the melflufen plus panobinostat combination induced high efficacy (mean DSS = 60) across all patient samples (*n* = 13) and with a significantly increased DSS for the triplet versus the double combination melflufen and dexamethasone (Fig. 5E). Interestingly, for the triple combination synergistic effects required low concentrations (0.1–10 nm for panobinostat and 0.1–10 nm for melflufen), similar to what was observed for the double combination (Fig. 5F).

In summary, these results indicated a clear synergistic activity between melflufen and panobinostat and future clinical investigation on use of the combination is warranted.

### 3.5 Synergy effects in triple combinations in MM cells

**Fig. 6:**
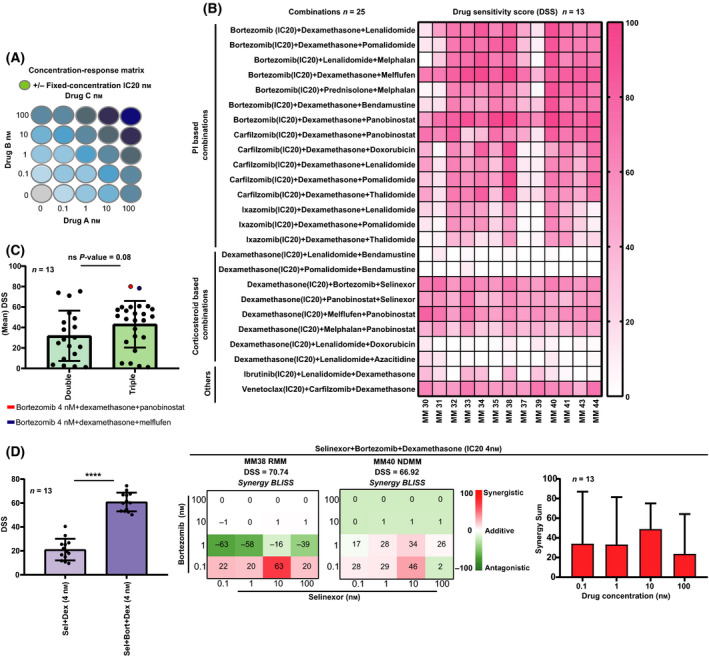
Triple combination *ex vivo* responses in MM samples. (A) Illustration of the concentration‐matrix designed to test a panel of triple combinations in MM cells from patients. Here, the two drugs (drug A plus drug B) were tested each at multiple concentrations, focusing only on the lower concentration range (0.1–100 nm), while a third drug (drug C) was utilized at its fixed IC_20_ concentration as indicated. (B) The MM cells isolated from BM samples (*n* = 13) were exposed to a selected panel of drugs used alone and in triple combinations and viability assessed as in Fig. 2. The heatmap shows estimated drug effects on MM cell viability represented as DSS for 25 triple combinations. (C) Comparison of drug sensitivity scores (mean DSS) between double and triple combinations in MM samples (*n* = 13, mean ± SD). (D) Comparison of drug sensitivity (DSS) from *n* = 13 patient samples between selinexor in combination with dexamethasone at 4 nm and the corresponding triple combination with bortezomib (Left plot, mean ± SD). Examples of 2D heatmap plots (Bliss method) show predicted synergistic (red) and antagonistic (green) effects for the triplet as indicated on MM cell viability for patient samples MM38 and MM40 (Middle plots). Bar plot shows synergy scores for the triplet calculated as synergy sum (see Materials and methods) for all the patient samples (*n* = 13, mean + SD) (right plot). Combinations with a Bliss score > 0 were considered synergistic. Significance was calculated using Mann–Whitney *U*‐test (**** denote *P* < 0.001).

To investigate the effects of triple combinations on MM cell viability, we performed drug sensitivity screening on MM cells (*n* = 13) (for assay setup, see Fig. 6A). Combinations with a similar mechanism, for example, PI‐based combinations, aligned with similar sensitivity (Fig. 6B). In line with clinical studies, the triple combination bortezomib plus dexamethasone plus lenalidomide was highly effective in almost all patient samples (median DSS 69). Interestingly, MM cells from a patient (MM39) refractory to the bortezomib plus dexamethasone plus lenalidomide combination showed low *ex vivo* sensitivity (DSS < 15) (Fig. 6B and Table S1), suggesting that *ex vivo* sensitivity can reflect clinical responses.

When comparing DSS between triple versus double combinations, a trend toward significant increases in efficacy was detected (Fig. 6C). The clinically approved bortezomib plus dexamethasone plus panobinostat (Fig. 6C, red) and the bortezomib plus dexamethasone plus melflufen (Fig. 6C, blue) combinations (the latter in phase I/II) induced the highest sensitivity (DSS = 80 and 78.3, respectively), when compared to the top‐ranked double combinations (Fig. 6C).

In MM, the triplet bortezomib plus selinexor plus dexamethasone as a novel treatment option has been reported [40, 41]. Here, we demonstrated that this triple combination was more effective than the corresponding double combination selinexor plus dexamethasone (Fig. 6D left plot). The triple combination of selinexor plus bortezomib plus dexamethasone induced synergistic effects at low concentrations, with the maximum synergistic effect at 0.1 nm bortezomib with 10 nm selinexor, for patient samples MM38 and MM40 (Fig. 6D, middle plots). Synergistic effects were also observed for all patient samples (*n* = 13) (Fig. 6D right plot).

In summary, our current pipeline has been able to incorporate triple combinations in *ex vivo* screening and predicted synergistic viability effects from complex concentration‐response matrices.

### 3.6 Correspondence between *ex vivo* drug sensitivity and clinical features

**Fig. 7:**
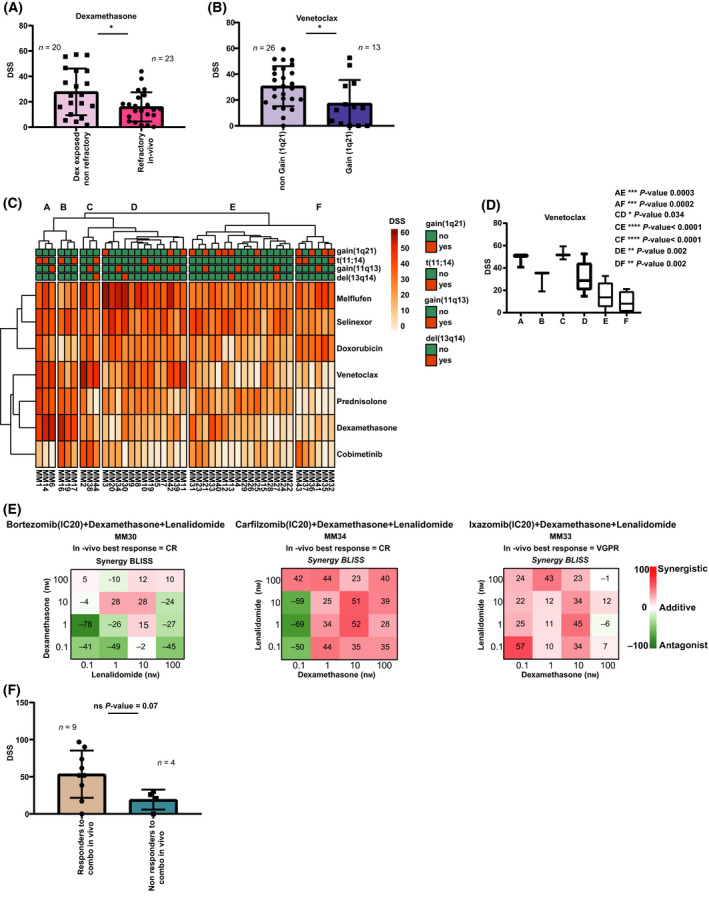
Correlation analysis of drug sensitivity profile versus patient clinical data. (A) *Ex vivo* drug sensitivity in dexamethasone nonrefractory (*n* = 20) versus *in vivo* refractory patient samples (*n* = 23) (mean ± SD, along with individual data). (B) *Ex vivo* drug sensitivity to venetoclax in patient samples lacking gain(1q21) (*n* = 26) compared to patient samples with gain(1q21) (*n* = 13) (mean ± SD, along with individual data). Significance was calculated using Mann–Whitney *U*‐test (* denotes *P* < 0.05). (C) Unsupervised hierarchical clustering analyses of drug sensitivities by DSS and FISH cytogenetics (annotation top, legend right) of MM samples (*n* = 44). The analyses identified six sample clusters (columns A–F). (D) Comparison of venetoclax sensitivity by cluster group. Note: decreased sensitivity for group E and F compared to cluster A, B, C, and D. Significant differences are denoted using one‐way ANOVA applying a Holm–Sidak’s multiple comparison test (* denotes *P* < 0.05, **P < 0.01, ***P < 0.001, ****P < 0.0001). (E) Examples of 2D synergy heatmaps from *n* = 3 patient samples illustrate synergistic effects for clinically approved triple combinations in MM, and *in vivo* best response to the indicated triple combinations, after the time of sampling (CR = complete response; VGPR = very good partial response). (F) Comparison between *ex vivo* sensitivity for combinations tested and patient samples (*n* = 13) classified as *in vivo* responders/non responders, following the International Myeloma Working Group Response Criteria. Bars with error indicate mean ± SD. (ns, not significant *P*‐value = 0.07 using Mann–Whitney *U*‐test).

Clinical features such as best response after the time of sampling together with cytogenetic data for each patient were used to gain further knowledge on subtle drug sensitivity patterns. Significantly increased sensitivity was observed in MM cells from nonrefractory patients exposed to dexamethasone (mean DSS 27.7), compared to MM cells from refractory patients (mean DSS 15.9) (Fig. 7A). In line with earlier studies [5], our screening identified heterogeneous responses to venetoclax and the presence of t(11;14) was associated with higher sensitivity to venetoclax (mean DSS 38.5 and 24.3, respectively), particularly in samples from patients with t(11;14) and lacking gain(1q21) (Fig. S3A). Intriguingly, we saw that MM cells from patients with gain(1q21) mutation had lower sensitivity to venetoclax compared to MM cells from patients without this mutation (mean DSS 17.4 and 30.7, respectively) (Fig. 7B).

Our clustering analysis confirmed that MM cells from patient subgroups enriched with gain(1q21) had a significantly decreased venetoclax sensitivity, compared to those without (Fig. 7C,D). These findings suggest that gain(1q21) should be further explored as a potential predictive marker of venetoclax sensitivity in MM.

Next, we compared combination data included in the screening with *in vivo* patient clinical responses (Table S1). Notably, patients tested with triple combinations (Fig. 7E) and classified as synergistic or sensitive (median DSS 51.8) by our screen (*n* = 3), reached a complete response (CR) or a VGPR to their current drug regimen. Altogether, patients classified as responders (*n* = 9) displayed a trend toward significance of increased *ex vivo* sensitivity (median DSS of 51.8), compared to patients achieving a poor response (*n* = 4) (median DSS of 23.4) [minimal response (MR) = 1, stable disease (SD) = 1, progressive disease (PD) = 2] (Fig. 7F).

Taken together our results could be linked to *in vivo* clinical responses, and thus, our pipeline shows a potential predictive impact to determine effective treatments.

## 4 Discussion

In the present study, we implemented *ex vivo* drug sensitivity assays to study drug responses and synergies for single drugs and combinations in MM cells. Special features of our *ex vivo* pipeline were the short‐term stimulation (48 h) of BMMCs, supporting MM cell *ex vivo* cultures prior to drug screening [10]. We present a fast and accurate protocol that enables intervention in a clinically suitable 5‐day time frame.

Distinct differential sensitivities to dexamethasone and venetoclax observed in our screen highlights the possibility for implementing precision medicine strategies in the treatment of MM. Dexamethasone treatment is widely applied in myeloma and resistance is common. As expected, decreased dexamethasone sensitivity in cells from refractory‐myeloma patients was demonstrated, in agreement with the clinical experience in these patients. This may help in optimizing the use of corticosteroids in MM and avoid dexamethasone when corticosteroids no longer benefit patients.

Consistent with earlier findings [5, 42, 43], we showed that the presence of gain(1q21) was associated with decreased sensitivity to the BCL‐2 inhibitor venetoclax. Gain(1q21) has been associated with increased expression of induced myeloid leukemia cell differentiation protein (MCL‐1), which is known to confer drug resistance to venetoclax [44, 45]. It appears from our data and those of others that gain(1q21) is an attractive predictive biomarker to improve treatment efficacy and to identify patients who could respond to venetoclax treatment in MM. In addition, it should be noticed that the response rate to venetoclax among patients with high BCL‐2 expression is as good as among those with t(11;14). The BCL‐2 group is generally significantly larger. Therefore, this may explain some responses in patients who do not have t(11;14) [46, 47].

We demonstrated that combinations combining standard‐of‐care myeloma drugs (e.g., PIs) with emerging agents such as panobinostat and melflufen were highly effective *ex vivo*. Interestingly, a low concentration of the priming drugs (i.e., IC_20_), was sufficient to exert a greater viability response for the double combinations compared to single drug. The use of a priming drug at a fixed concentration in lieu of a full concentration matrix for combinations allowed us to save patient material, while still being able to predict synergistic responses also outside the tested concentration ranges. Overall, we noticed that bortezomib and carfilzomib used in double and triple combinations were able to potentiate dexamethasone and selinexor effects on viability, identifying beneficial effects of PIs as sensitizing agents in combination treatments.

We uncovered new synergistic anti‐myeloma effects between melflufen and panobinostat. This may indicate that panobinostat could sensitize MM cells to melflufen activity providing a stronger combination response when compared to single melflufen activity. Importantly, the synergistic effect observed between melflufen plus panobinostat at low drug concentrations may justify a lower drug dose of either drug in patients, which perhaps could improve the panobinostat toxicity profile clinically. Panobinostat and melflufen have so far been investigated mostly in combination with PIs and dexamethasone. While adverse events and low clinical activity have recently been reported for these two drugs, it is still not clear which patients will benefit from these treatments. Our approach may help predicting which patients will most likely respond to the effect of this combination treatment. Generally, panobinostat is administered at 10–20 mg when in combination with bortezomib. Clinical data have reported that panobinostat when administered at 20 mg, in combination with bortezomib, gives a higher response rate; however when administered at 10 mg it gives a better tolerability [48]. The dose 20 mg corresponds to an *in vitro* concentration of approximately 20–40 nm. Melflufen is commonly administered at 50 mg [49], which gives an *in vitro* concentration > 300 nm. Our data demonstrated that synergy could be already achieved with panobinostat at 8 nm combined with melflufen < 10 nm *ex vivo* (Fig. 5). The synergy between panobinostat and melflufen observed in our study was achieved at a lower concentrations compared to those used in the clinic with no loss of activity, which may improve clinical response and tolerability profiles of these treatments in MM. Both drugs have recently been removed from the US market, while panobinostat is available and melflufen under consideration, in Europe. The results presented here might give interest to follow‐up on the novel synergistic efficacy of panobinostat–melflufen combined treatment *in vivo* studies and define better predictive biomarkers and clinical signatures that would help selecting patients in future precision medicine clinical trials.

Samples with higher sensitivity to the melflufen plus panobinostat combination were characterized by the presence of cell cycle genetic aberrations such as del(13q14) and gain(11q13), known to affect the expression of the RB1 and CCND1 genes, respectively.

This may reflect an increased proliferation rate of these patient’s MM cells as described [50, 51] that may explain the increased *ex vivo* sensitivity to the melflufen plus panobinostat combination. Future studies should examine the exact role these mutations play with respect to melflufen and panobinostat treatment.

## 5 Conclusions

In summary, we provide a method for rapid assessment of synergy‐linked drug sensitivities combining multiple agents in patient‐derived MM cells. However, limitations need to be addressed in future studies. More data are needed for correlation of *ex vivo* testing with clinical drug responses. *Ex vivo* synergy testing should be considered as a method to predict optimal dosing of regimes with proven clinical activity for use in some patients, which we hope will reduce unnecessary toxicities. Consistent with prior data [5, 7], we found that most patient samples in our screen displayed little sensitivity to IMIDs. It could be that these agents primarily exert their anti‐myeloma effects indirectly via micro‐environmental effects that should be considered in future designs of the drug sensitivity assays. As the amount of possible new combinations will also increase in MM, we hope that our work can provide utility to guide clinical decisions and to point out new possible efficacies, which can be further exploited in clinical trials.

## Conflict of interest

The authors declare no conflict of interest.

### Peer Review

The peer review history for this article is available at https://publons.com/publon/10.1002/1878‐0261.13191.

## Author contributions

KT designed the research together with LAM and GET, MG and DBTGR designed methodology, performed the experiments and analyzed the data together with AG, SSS, AC, LAM, and KT. FS and GET contributed with patient samples, clinical data and interpreted data. MG wrote the paper with input from KT. All authors read and commented on draft versions of the paper and approved the final version.

## Supporting information


**Fig. S1.** *Ex vivo* drug sensitivity screening is reproducible.

**Fig. S2.** Viability of CD138^+^ MM cells isolated from BMMC samples and the SK‐MM2 cell line after *in vitro* stimulation.

**Fig. S3.** *Ex vivo* drug sensitivity to venetoclax in MM patient samples versus specific cytogenetic characteristics.

**Table S1.** Patient clinical data.

**Table S2.** Single drug library used in the study.

**Table S3.** Double drug combinations used in the study on MM cells from 13 patient samples.

**Table S4.** Triple‐drug combinations used in the study on MM cells from 13 patient samples.

Supplementary Material

## Data Availability

The data that support the findings of this study are available from the corresponding author kjetil.tasken@medisin.uio.no upon reasonable request.
