# Supplementary figures and images for: Ex vivo drug sensitivity screening in multiple myeloma identifies drug combinations that act synergistically
Source: Mol Oncol. 2022 Mar 12;16(6):1241–58. doi: 10.1002/1878-0261.13191 (PMC8936517; doi:10.1002/1878-0261.13191)

Supplementary Figure 1

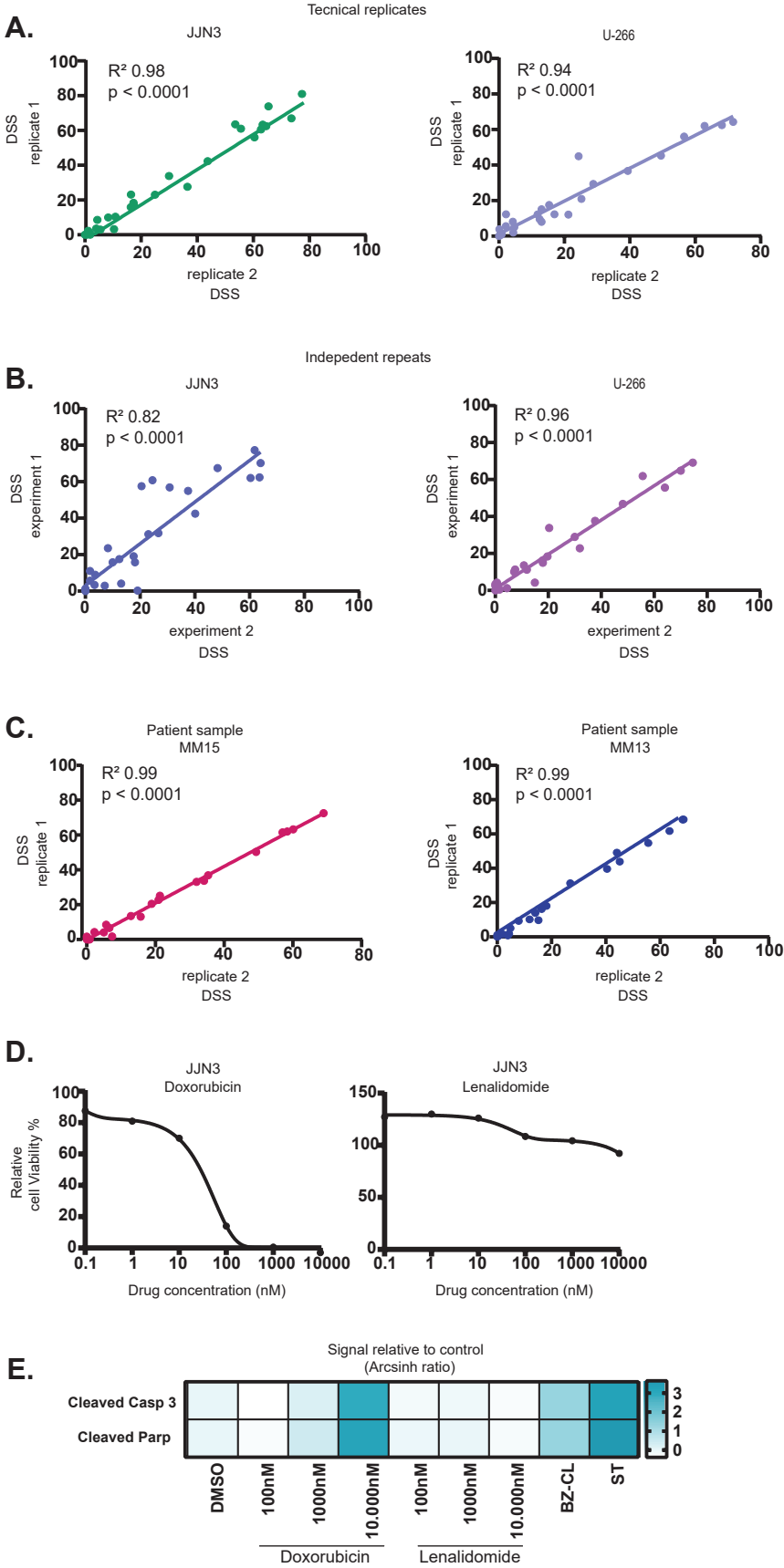

Supplement: Supplementary file 1 — Fig. S1. Ex vivo drug sensitivity screening is reproducible. [file MOL2-16-1241-s008.pdf]

Supplementary Figure 2

A.

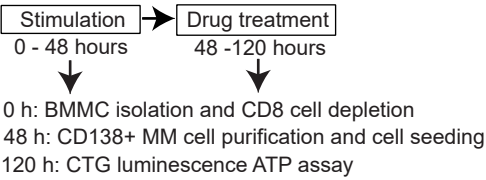

B.

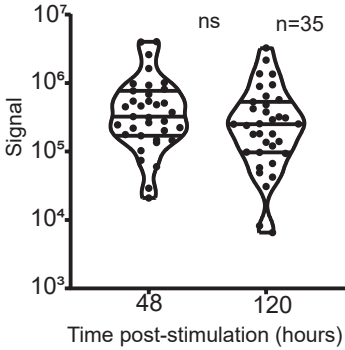

C.

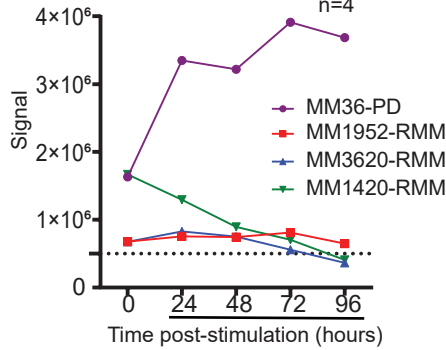

D.

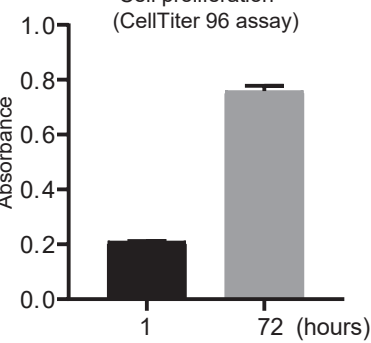

E.

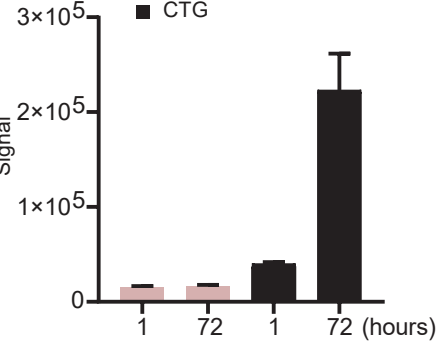

Supplement: Supplementary file 2 — Fig. S2. Viability of CD138+ MM cells isolated from BMMC samples and the SK‐MM2 cell line after in vitro stimulation. [file MOL2-16-1241-s001.pdf]

Supplementary Figure 3

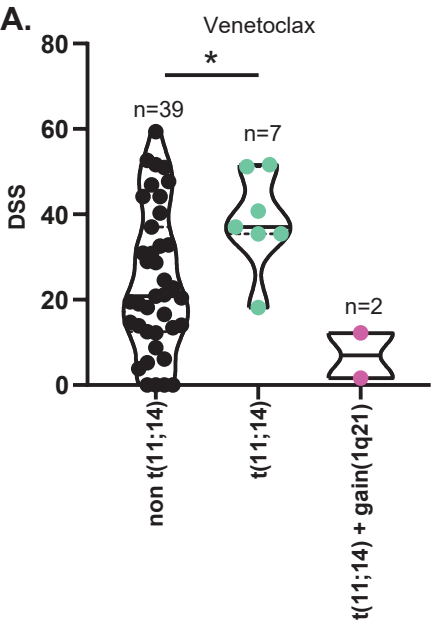

Supplement: Supplementary file 3 — Fig. S3. Ex vivo drug sensitivity to venetoclax in MM patient samples versus specific cytogenetic characteristics. [file MOL2-16-1241-s004.pdf]
