# Supplementary material for: Ex vivo drug sensitivity screening in multiple myeloma identifies drug combinations that act synergistically
Source: Mol Oncol. 2022 Mar 12;16(6):1241–58. doi: 10.1002/1878-0261.13191 (PMC8936517; doi:10.1002/1878-0261.13191)
Supplement: Supplementary file 4 — Table S1. Patient clinical data. [file MOL2-16-1241-s006.pdf]

Supplementary Table S1. Patient clinical data (n=44)

Column1	Patient ID	Diagnosis at time of sampling	Drug screening type	FISH (Fluorescence In Situ Hybridization)	ISS stage*	Treatment after sampling*	Best Response after sampling*
1	MM3	RMM	single drug screen	t(14;16), gain(1q21)	II	melflu+dex	PD
2	MM5	RMM	single drug screen	gain(11q13)	II	ixa+dex	PR
3	MM1	RMM	single drug screen	del13, t(11;14)	I	cob+ven+atezol	PR
4	MM2	RMM	single drug screen	Normal	I	len+dex	CR
5	MM15	RMM	single drug screen	Normal	II	pano+bort+dex	MR
6	MM4	RMM	single drug screen	gain(11q13)	II	carf+dex+cyclo+ASCT	VGPR
7	MM11	RMM	single drug screen	gain(1q21),	Unknown	ixa+dex	MR
8	MM9	NDMM	single drug screen	t(4;14)	I	bort+len+dex+ASCT+carf	sCR/MRDneg
9	MM6	NDMM	single drug screen	gain(11q13), gain(6p21)	II	cyclo+bort+dex+ASCT	VGPR
10	MM16**	RMM	single drug screen	t(11;14)	II	same as MM17	
11	MM17**	RMM	single drug screen	t(11;14)	II	carf+dex	PD
12	MM21	RMM	single drug screen	gain(1q21), del(1p32), gain(11q13), del(14q32)	I	cob+ven+atezol	PD
13	MM19	RMM	single drug screen	Not available	II	bort+dex	VGPR
14	MM7	RMM	single drug screen	t(4;14)	II	pom+dex	MR
15	MM12	SMM	single drug screen	gain(1q21), del(14q)	II	len+dex	VGPR
16	MM8	RMM	single drug screen	Normal	I	carf+dex	PR
17	MM13	NDMM	single drug screen	del(13q14), gain(1q21)	I	bort+len+dex	CR
18	MM20	RMM	single drug screen	gain(1q21) negative	I	cob+ven+atezol	PD
19	MM10	SMM	single drug screen	t(11;14)	I	not treated	
20	MM14	RMM	single drug screen	t(11;14)	Unknown	len+dex	VGPR
21	MM22	RMM	single drug screen	Not available	II	missing info on treatment	MR
22	MM23	RMM	single drug screen	Not available	II	ixa+dex	CR
23	MM24	RMM	single drug screen	Normal	II	cob+ven+atezol	PR
24	MM25	RMM	single drug screen	gain(1q21), gain(1p32), gain(11q13), gain(6p21), gain(MAFB)20q12, del17(TP53)	III	melflu+dex	PD
25	MM26	RMM	single drug screen	del 17(TP53),del MAF(20q13)	II	melflu+dex	VGPR
26	MM27*	RMM	single drug screen	Del(13q14), del(MAF)16q23, gain(11q13)	Unknown	pom+dex	SD
27	MM19*	RMM	single drug screen	Normal	II	pano+bort+dex	PD
28	MM28	RMM	single drug screen	Normal	I	melflu+dex	MR
29	MM29	RMM	single drug screen	Not available	II	ixa+dex	PR
30	MM44	RMM	single drug screen	t(11;14), del (13q14)	III	missing info on treatment	
31	MM36	RMM	single drug screen	t(4;14), gain(1q21)	I	dara+pom+dex	PD
32	MM30	NDMM	combinatorial screen	del(13q14), del(16q23), del(20q12)	II	dara+bort+len+dex+ASCT	sCR
33	MM31	RMM	combinatorial screen	gain(1q21), t IGH/FGR3 t(4;14)(P16q;32), gain (17p13), del13q	I	pom+dex	PR
34	MM32	RMM	combinatorial screen	gain(1q21), t(11;14)(q13q32), del13q, gain FGR3 (4p16)	I	pom+dex	PD
35	MM33	NDMM	combinatorial screen	Normal	I	ixa+len+dex+ASCT	VGPR
36	MM34	NDMM	combinatorial screen	gain (11q13)	I	carf+len+dex	sCR
37	MM35	RMM	combinatorial screen	gain(1q21), del (17p13) (TP53), del14q, del4p, del16q (CCND3), gain (6p21)	I	dara+bort+dex	PR
38	MM40	NDMM	combinatorial screen	gain (1q21), del (1p32), t(14;16)(q32;q;23),gain (4p16), gain (6p21), gain (11q13), gain (20q12)	III	bort+len+dex	VGPR
39	MM37	RMM	combinatorial screen	t(11;14)	Unknown	ven+dex	MR
40	MM42	RMM	combinatorial screen	gain(1q21), del(1p32), gain(11q13), gain(14q32), t(14:20)	I	carf+dex	PR
41	MM39	RMM	combinatorial screen	gain (11q13), del (16q23), del17 (TP53), del13q	I	bor+len+dex, ASCT+len+dex; melflu+dex	SD
42	MM41	NDMM	combinatorial screen	gain(14p16), gain(11q13)	II	ixa+len+dex+ASCT	PR
43	MM38	RMM	combinatorial screen	gain(14q23), gain(11q13)	Unknown	melflu+dex	PD
44	MM43	RMM	combinatorial screen	gain (1q21), t(11:14)	I	missing info on treatment	PD

Comments:

Treatment:
Carf: carfilzomib; Cyclo: cyclophosphamide; Bort:bortezomib; Dex:dexamethasone; Len:lenalidomide; ASCT: Autologous Stem Cell Transplant; Melflu: melflufen; Dara: daratumab: Atezol: atezolizumab; Ixa: ixazomib; Pano:panobinostat; Pom: pomalidomide; Ven: venetoclax; Cob: cobimetinib.

Best Response* based on International Myeloma Working Group Response Criteria:
MRDneg: minimal residual disease negative, sCR:stringent complete response; CR: complete response, VGPR: very good partial response; PR: partial response; MR: minimal response; SD: standard disease; PD: progressive disease.

Disease stage/Diagnosis*:
NDMM:newly diagnosed multiple myeloma; RMM: relapsed multiple myeloma; SMM: smoldering multiple myeloma.

ISS stage*: International Staging System for MM (based on Serum β2 microglobilin and Serum Albumin)
ISS stage I: defined as serum β2-microglobulin level less than 3.5 mg/L and serum level > 3.5 g/dL;
ISS stage II: included all patients with neither stage I nor stage III disease
ISS stage III: defined as serum β2-microglobulin level > 5.5 mg/L, irrespective of serum albumin level.

* = same patient
** = same patient
