# Supplementary material for: Ex vivo drug sensitivity screening in multiple myeloma identifies drug combinations that act synergistically
Source: Mol Oncol. 2022 Mar 12;16(6):1241–58. doi: 10.1002/1878-0261.13191 (PMC8936517; doi:10.1002/1878-0261.13191)
Supplement: Supplementary file 5 — Table S2. Single drug library used in the study. [file MOL2-16-1241-s002.pdf]

Supplementary Table S2. Single drug library used in the study

Drug name	Vendor	Class/mechanism	Target/mechanism	Min. Conc. Tested nM	Max. Conc. Tested nM
Acalabrutinib	selleckchem	kinase inhibitor	BTK	0.1	10000
AT9283	selleckchem	kinase inhibitor	Aurora A/B; JAK2/3	0.1	10000
Azacitidine	selleckchem	chemotherapeutic	DNA methylation	0.1	10000
Bendamustine HCl	selleckchem	alkylating agent	DNA-damaging	0.1	10000
Bortezomib	selleckchem	proteasome inhibitor	ProteasomeB5/Reversible, Protein stability/NF-KB	0.1	10000
Carfilzomib	selleckchem	proteasome inhibitor	ProteasomeB5/Irreversible, Protein stability/NF-KB	0.1	10000
Cisplatin	selleckchem	chemotherapeutic	DNA	0.1	10000
Clarithromycin	selleckchem	chemotherapeutic	DNA	0.1	10000
Cobimetinib	selleckchem	kinase inhibitor	MEK1/2	0.1	10000
Cyclophosphamide	selleckchem	alkylating agent	Unmetabolized	0.1	10000
Dabrafenib	selleckchem	kinase inhibitor	RAF1, BRAF	0.1	10000
Dexamethasone	selleckchem	immunosuppressive	Interleukin receptor	0.1	10000
Doxorubicin	selleckchem	chemotherapeutic	DNA	0.1	10000
Etoposide	selleckchem	chemotherapeutic	Topoisomarese II	0.1	10000
Ibrutinib	selleckchem	kinase inhibitor	BTK	0.1	10000
Ixazomib	selleckchem	proteasome inhibitor	ProteasomeB5/Reversible, Protein stability better selectivity	0.1	10000
Lenalidomide	selleckchem	immunomodulator	CRBN, immunomodulation	0.1	10000
Melflufen	selleckchem	peptide-conjugated alkylator	DNA-damaging	0.1	10000
Melphalan	selleckchem	alkylating agent	DNA-damaging	0.1	10000
Oprozomib	selleckchem	proteasome inhibitor	ProteasomeB5/Irreversible, Protein stability	0.1	10000
Panobinostat	selleckchem	HDAC inhibitors	HDAC1, HDAC2, HDAC3, HDAC4, HDAC6, HDAC7, HDAC8, HDAC9	0.1	10000
Pomalidomide	selleckchem	immunomodulator	CRBN, immunomodulation	0.1	10000
Prednisolone	selleckchem	immunosuppressive	Immunosuppressant	0.1	10000
Romidepsin	selleckchem	HDAC inhibitors	HDAC1, HDAC2, HDAC3, HDAC4, HDAC5, HDAC6, HDAC7, HDAC8, HDAC9	0.1	10000
Selinexor	selleckchem	small molecule inhibitor	XPO1	0.1	10000
Simvastatin	selleckchem	small molecule inhibitor	HMG-CoA reductase	0.1	10000
Thalidomide	selleckchem	immunomodulator	CRBN, immunomodulation	0.1	10000
Vemurafenib	selleckchem	kinase inhibitor	RAF1, BRAF	0.1	10000
Venetoclax	selleckchem	apoptosis inducer	BCL-2	0.1	10000
Vorinostat	selleckchem	HDAC inhibitors	HDAC1, HDAC10, HDAC11, HDAC2, HDAC3, HDAC5, HDAC6, HDAC8, HDAC9	0.1	10000
