# Supplementary material for: Ex vivo drug sensitivity screening in multiple myeloma identifies drug combinations that act synergistically
Source: Mol Oncol. 2022 Mar 12;16(6):1241–58. doi: 10.1002/1878-0261.13191 (PMC8936517; doi:10.1002/1878-0261.13191)
Supplement: Supplementary file 6 — Table S3. Double drug combinations used in the study on MM cells from 13 patient samples. [file MOL2-16-1241-s005.pdf]

Supplementary Table S3.Double drug combinations used in the study on MM cells from 13 patient samples

Drug name (IC20 nM)	Second drug name	Min. Conc. Tested nM	Max. Conc. Tested nM	IC20 nM concentration	Study Phase	Clinical Trials ID
Bortezomib(IC20)	dexamethasone	0.1	1000	4	III	NCT01568866
Bortezomib(IC20)	selinexor	0.1	1000	4		
Carfilzomib(IC20)	dexamethasone	0.1	1000	3	II	NCT01568866, NCT03512353
Ixazomib(IC20)	pomalidomide	0.1	1000	11		
Dexamethasone(IC20)	ixazomib	0.1	1000	4	II	NCT03170882
Dexamethasone(IC20)	oprozomib	0.1	1000	4	I/II	NCT01832727
Dexamethasone(IC20)	melflufen	0.1	1000	4	I/II	NCT01897714, NCT02963493
Dexamethasone(IC20)	melphalan	0.1	1000	4	III	NCT01078454
Dexamethasone(IC20)	bendamustine	0.1	1000	4	II	NCT01222260
Dexamethasone(IC20)	lenalidomide	0.1	1000	4	III	NCT00689936
Dexamethasone(IC20)	pomalidomide	0.1	1000	4	II	NCT01946477
Dexamethasone(IC20)	selinexor	0.1	1000	4	II	NCT02336815
Dexamethasone(IC20)	venetoclax	0.1	1000	4	II	NCT01794520
Prednisolone(IC20)	melphalan	0.1	1000	150		
Panobinostat(IC20)	melflufen	0.1	1000	8		
Panobinostat(IC20)	selinexor	0.1	1000	8		
Melflufen(IC20)	pomalidomide	0.1	1000	50		
Melflufen(IC20)	prednisolone	0.1	1000	50		
Venetoclax(IC20)	cobimetinib	0.1	1000	21	I/II	NCT03312530
