# Supplementary material for: Ex vivo drug sensitivity screening in multiple myeloma identifies drug combinations that act synergistically
Source: Mol Oncol. 2022 Mar 12;16(6):1241–58. doi: 10.1002/1878-0261.13191 (PMC8936517; doi:10.1002/1878-0261.13191)
Supplement: Supplementary file 7 — Table S4. Triple‐drug combinations used in the study on MM cells from 13 patient samples. [file MOL2-16-1241-s003.pdf]

Supplementary Table S4.Triple drug combinations used in the study on MM cells from 13 patient samples

Drug name (IC20 nM)	First drug name	Second drug name	Min. Conc. Tested nM	Max. Conc. Tested nM	IC20 nM concentration	Study Phase	Clinical Trials ID
Bortezomib	Dexamethasone	Lenalidomide	0.1	100	4	II/	NCT00378209/NCT01782963
Bortezomib	Dexamethasone	Pomalidomide	0.1	100	4	III	NCT01734928
Bortezomib	Lenalidomide	Melphalan	0.1	100	4		
Bortezomib	Dexamethasone	Melflufen	0.1	100	4	I/II	NCT03481556
Bortezomib	Prednisolone	Melphalan	0.1	100	4	II	NCT00734149
Bortezomib	Dexamethasone	Bendamustine	0.1	100	4	II	NCT02224729
Bortezomib	Dexamethasone	Panobinostat	0.1	100	4	II	NCT02654990
Carfilzomib	Dexamethasone	Panobinostat	0.1	100	3	I	NCT01549431
Carfilzomib	Dexamethasone	Doxorubicin	0.1	100	3	I/II	NCT01246063
Carfilzomib	Dexamethasone	Lenalidomide	0.1	100	3	III	NCT01080391
Carfilzomib	Dexamethasone	Pomalidomide	0.1	100	3	I/II	NCT01464034
Carfilzomib	Dexamethasone	Thalidomide	0.1	100	3	II	NCT03140943
Ixazomib	Dexamethasone	Lenalidomide	0.1	100	11	II	NCT02253316
Ixazomib	Dexamethasone	Pomalidomide	0.1	100	11	I/II	NCT04094961
Ixazomib	Dexamethasone	Thalidomide	0.1	100	11	II	NCT02410694
Dexamethasone	Lenalidomide	Bendamustine	0.1	100	4	I/II	NCT01049945
Dexamethasone	Pomalidomide	Bendamustine	0.1	100	4	I/II	NCT01754402
Dexamethasone	Bortezomib	Selinexor	0.1	100	4	III	NCT02343042, NCT03110562
Dexamethasone	Panobinostat	Selinexor	0.1	100	4		
Dexamethasone	Melflufen	Panobinostat	0.1	100	4		
Dexamethasone	Melphalan	Panobinostat	0.1	100	4	I/II	NCT00743288
Dexamethasone	Lenalidomide	Doxorubicin	0.1	100	4	I/II	NCT00306813
Dexamethasone	Lenalidomide	Azacitidine	0.1	100	4	I/II	NCT01155583
Ibrutinib	Lenalidomide	Dexamethasone	0.1	100	3000	I	NCT03015792
Venetoclax	Carfilzomib	Dexamethasone	0.1	100	21	II	NCT02899052
