# Supplementary material for: Ex vivo drug sensitivity screening in multiple myeloma identifies drug combinations that act synergistically
Source: Mol Oncol. 2022 Mar 12;16(6):1241–58. doi: 10.1002/1878-0261.13191 (PMC8936517; doi:10.1002/1878-0261.13191)
Supplement: Supplementary file 8 — Supplementary Material [file MOL2-16-1241-s007.docx]

**SUPPLEMENT TO**

***Ex vivo* drug sensitivity screening in multiple myeloma identifies drug combinations that act synergistically**

Mariaserena Giliberto^1^**^,^**^2,5^ , Deepak B. Thimiri Govinda Raj^1,2,5^, Andrea Cremaschi^5,6^, Sigrid S Skånland^1,2,5^ Alexandra Gade^5^, Geir E Tjønnfjord^2,4^, Fredrik Schjesvold^2,4,3^, Ludvig A Munthe^2,3^, and Kjetil Taskén^1,2,5,*^

**SUPPLEMENTARY FIGURE LEGENDS**

**Supplementary Figure 1. *Ex vivo* drug sensitivity screening is reproducible**

The experimental reproducibility between independent repeats and technical replicates for the single drug screen was assessed in MM cell lines (JJN3, U-266) and patient samples. The MM cells were exposed to 30 single drugs (see Supplementary Table S2) at 6 concentrations (0.1-10,000nM) for 72 hours, the cell viability was measured by CellTiter-Glo. **A-C** Linear correlation between DSS in two technical repeats (**A**), independent experiments (**B**) performed on two MM cell lines (left and right columns) and between technical repeats in two patient samples (**C**). **D-E** Concentration-responses for the effects of doxorubicin and lenalidomide on JJN3 cell viability in the CellTiter-Glo assay (**D**), and on the expression of apoptosis markers by flow cytometry (**E**), MM cells were exposed to doxorubicin and lenalidomide for 72 hours at indicated concentrations. Cells were then fixed, permeabilized and stained with indicated intracellular antibodies for flow analysis (see “Materials and Method”). The median fluorescence intensity for the specific apoptotic antibodies is normalized to the signal of an isotype control and depicted as arcsinh ratio. DSS, drug sensitivity score.

**Supplementary Figure 2. Viability of CD138^+^ MM cells isolated from BMMC samples and the SK-MM2 cell line after *in vitro* stimulation**

**A** Workflow of the experiments presented in panel B. BMMC; bone marrow mononuclear cells, CTG; CellTiter-Glo.

**B** End-point measurements for the CellTiter-Glo luminescence ATP assay indicate viability at 48 hours (left) and 120 hours (right) for patient-derived CD138^+^ MM cells (n=35) in DMSO controls (0.1%). Lines in the violin plots show median and quartiles. ns, not significant using Mann Whitney U-test.

**C** Viability at different time-points (0 - 96 hours) for patient-derived CD138^+^ MM cells (n=4) by the CellTiter-Glo luminescence ATP assay. Each line is color coded for the corresponding patient sample. The purple line indicates MM cells from a patient (MM36) with a progressive disease (PD) status. Each viability point represents the mean of seven (0h) or fourteen replicates (24 – 96h) in DMSO (0.1%) treated samples. RMM; relapsed multiple myeloma.

**D** Cell proliferation of the SK-MM2 cell line was assessed after 1 and 72 hours using the CellTiter 96 AQueous One Solution Cell Proliferation Assay. Values represent the mean of five replicates ± standard deviation.

**E** Signal values for the indicate cell viability for SK-MM2 cell line after 1 and 72 hours by CellTox-Green (left) and CellTiter-Glo (right) assays. MM2 cells (5000 cells per well in 25 μl volume per well) were pre-seeded in DMSO control (0.1%). Values represent the mean of five replicates ± standard deviation.

**Supplementary Figure 3. *Ex vivo* drug sensitivity to venetoclax in MM patient samples versus specific cytogenetic characteristics**

**A** *Ex vivo* drug sensitivity to venetoclax in patient samples lacking t(11;14) (n=39) compared to patient samples with t(11;14) (n=7), as well as samples with both gain(1q21) and t(11;14) (n=2). Differences between samples were assessed using Mann Whitney U-test (* denotes P < 0.05). DSS, drug sensitivity score.

**SUPPLEMENTARY TABLE LEGENDS**

**Table S1.** Patient clinical data.

**Table S2.** Single drug library used in the study.

**Table S3.** Double drug combinations used in the study on MM cells from 13 patient samples.

**Table S4.** Triple drug combinations used in the study on MM cells from 13 patient samples.
